# Abundance of live ^244^Pu in deep-sea reservoirs on Earth points to rarity of actinide nucleosynthesis

**DOI:** 10.1038/ncomms6956

**Published:** 2015-01-20

**Authors:** A. Wallner, T. Faestermann, J. Feige, C. Feldstein, K. Knie, G. Korschinek, W. Kutschera, A. Ofan, M. Paul, F. Quinto, G. Rugel, P. Steier

**Affiliations:** 1 Department of Nuclear Physics, Australian National University, Canberra, Australian Capital Territory 0200, Australia; 2 VERA Laboratory, Faculty of Physics, University of Vienna, Währinger Strasse 17, A-1090 Vienna, Austria; 3 Physik Department, Technische Universität München, D-85747 Garching, Germany; 4 Racah Institute of Physics, Hebrew University, Jerusalem 91904, Israel; 5 GSI Helmholtz-Zentrum für Schwerionenforschung GmbH, Planckstrasse 1, 64291 Darmstadt, Germany

## Abstract

Half of the heavy elements including all actinides are produced in *r*-process nucleosynthesis, whose sites and history remain a mystery. If continuously produced, the Interstellar Medium is expected to build-up a quasi-steady state of abundances of short-lived nuclides (with half-lives ≤100 My), including actinides produced in *r*-process nucleosynthesis. Their existence in today’s interstellar medium would serve as a radioactive clock and would establish that their production was recent. In particular ^244^Pu, a radioactive actinide nuclide (half-life=81 My), can place strong constraints on recent *r*-process frequency and production yield. Here we report the detection of live interstellar ^244^Pu, archived in Earth’s deep-sea floor during the last 25 My, at abundances lower than expected from continuous production in the Galaxy by about 2 orders of magnitude. This large discrepancy may signal a rarity of actinide *r*-process nucleosynthesis sites, compatible with neutron-star mergers or with a small subset of actinide-producing supernovae.

## Abstract (web summary)

The build-up of short-lived nuclides in the interstellar medium tells us about production frequency and yield of heavy elements by nucleosynthesis. Wallner *et al*. find a low abundance of live interstellar 244Pu detected from the deep-sea floor, suggesting a rarity for r-process nucleosynthesis sites.

## Graphical abstract

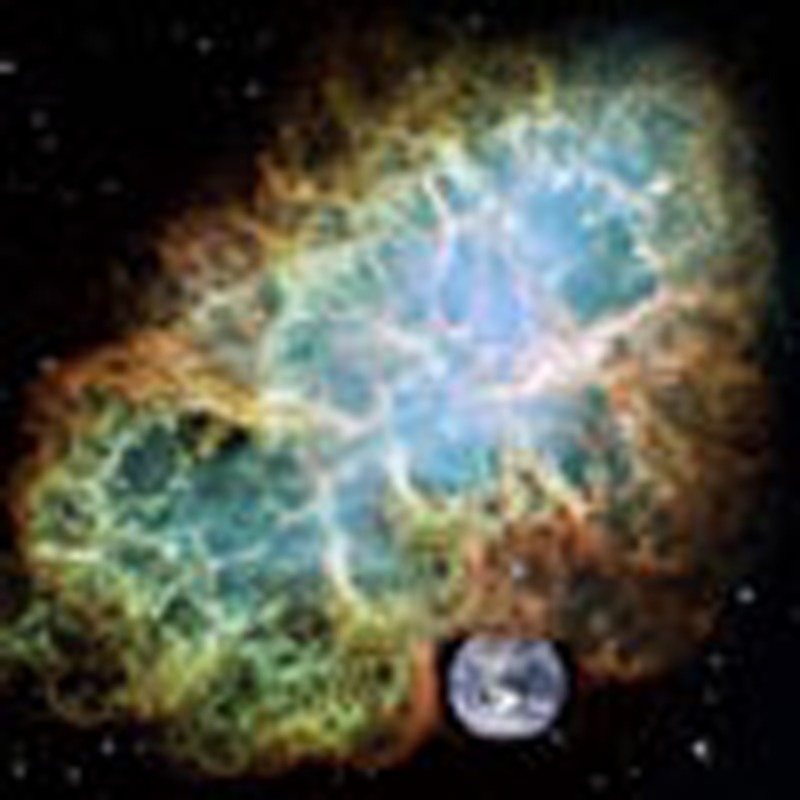

About half of all nuclides existing in nature and heavier than iron are generated in stellar explosive environments. Their production requires a very short and intense burst of neutrons (rapid neutron capture or *r*-process)^1^,^2^,^3^. The nuclides are formed via successive neutron captures on seed elements, following a path in the very neutron-rich region of nuclei. However, the relevant astrophysical sites, with supernovae (SN)^1^,^2^ and neutron-star (NS-NS) mergers^3^,^4^ as prime candidates, and the history of the *r*-process during the Galactic chemical evolution are largely unknown. The interstellar medium (ISM) is expected to become steadily enriched with fresh nucleosynthetic products and may also contain continuously produced short-lived nuclides (with half-lives ≤100 My (ref. 5)), including actinides produced in *r*-process nucleosynthesis.

Recent *r*-process models within SNe-II explosions, based on neutrino wind scenarios^6^,^7^, suffer difficulties on whether heavy elements can really be produced in these explosions. An alternative site is NS ejecta, for example, NS-NS or NS black-hole (NS-BH) mergers. Candidates of such neutron-star binary systems have been detected^8^,^9^. Estimations of an NS-NS merger event rate of about (2–3) × 10^−5^ per year in our galaxy would allow for such mergers to account for all heavy *r*-process matter in our Galaxy^3^,^10^,^11^.

It was pointed out by Thielemann *et al*.^4^ that observations of old stars indicate a probable splitting of the *r*-process into (i) a rare event that reproduces the heavy *r*-process abundances including actinides always in solar proportions, and (ii) a more frequent event responsible for the lighter *r*-process abundances. Galactic chemical evolution models^10^,^12^,^13^ show that NS mergers, occurring at late time in the life of a galaxy, cannot account for all the *r*-process nuclei found in very old stars^12^. Thus, recent models suggest different *r*-process scenarios (similar to *s* process), which might occur at different nucleosynthesis sites^3^,^13^.

To summarize, very few hints on astrophysical sites and galactic chemical evolution exist. First, the relative abundance distribution observed spectroscopically in a few old stars for *r*-process elements between barium and hafnium is very similar to that of the Solar System (SS)^1^,^14^, pointing to an apparently robust phenomenon; a large scatter for the *r*-process elements beyond Hf and also below barium is, however, observed^3^,^11^,^15^. Second, the early SS (ESS) is known to have hosted a set of short-lived radioactive nuclides (*t*_1/2_<~100 My)^5^,^16^,^17^,^18^, among them pure *r*-process nuclei such as ^244^Pu (half-life=81 My) and ^247^Cm (15.6 My) clearly produced no more than a few half-lives before the gravitational collapse of the protosolar nebula^19^,^20^,^21^,^22^,^23^.

We report here on a search for live ^244^Pu (whose abundance in the ESS relative to ^238^U was ~0.8% (see refs 5, 19, 20, 21, 23, 24, 25)) in deep-sea reservoirs, which are expected to accumulate ISM dust particles over long time periods. Our findings indicate that SNe, at their standard rate of ~1–2/100 years in the Galaxy, did not contribute significantly to actinide nucleosynthesis for the past few hundred million years. A similar conclusion is drawn, when related to the recent SNe history in the local interstellar environment: we do not find evidence for live ^244^Pu that may be locked in the ISM in accumulated swept-up material and that was transported to Earth by means of recent SNe activity. Our results suggest that actinide nucleosynthesis, as mapped through live ^244^Pu, seems to be very rare.

## Results

### Experimental concept

ISM dust particles^26^,^27^, assumed to be representative of the ISM, are known to enter the SS and are expected to reach and accumulate on Earth in long-term natural depositories such as deep-sea hydrogenous iron-manganese (FeMn) encrustations and sediments. Such a process is confirmed by inclusion in these archives of meteoritic ^10^Be, cosmogenic ^53^Mn and live ^60^Fe, the latter attributed to the direct ejecta of a close-by SN (refs 28, 29, 30). ^244^Pu-detection would be the equivalent for *r*-process nuclides of the γ-ray astronomy observations of live radioactivities^17^ produced by explosive nucleosynthesis in single SN events (for example, ^56^Ni (6.1 d), ^56^Co (77.3 d), ^44^Ti (60.0 y) or diffuse in the Galactic plane such as ^26^Al (0.72 My) and ^60^Fe (2.62 My), owing to their longer half-life).

Several models, based on the frequency of SN events, the nucleosynthesis yield and the radioactive half-life, were developed to calculate the abundance of ^244^Pu in quasi-secular equilibrium between production and radioactive decay rates. These models together with the flux and average mass of ISM dust particles into the inner SS measured by space missions in the last decade (Galileo, Ulysses, Cassini)^31^ are used here to estimate the corresponding influx of ^244^Pu nuclei onto Earth.

We compare our results also with a possible imprint of recent actinide nucleosynthesis (<15 My) from the SNe history of the Local Bubble (LB, a cavity of low density and hot temperature of ~200 pc diameter). Recent ISM simulations suggest about 14–20 SN explosions within the last 14 My^32^,^33^,^34^ that were responsible for forming the local ISM structure and the LB. ^244^Pu decay can be considered negligible during this period. The SN ejecta shaped the ISM and also accumulates swept-up material including pre-existing ^244^Pu from nucleosynthesis events prior to the formation of the LB^35^,^36^.

With a growth rate of a few millimetres per million years^37^, hydrogenous crusts will strongly concentrate elements and particles present in the water column above. The higher accumulation rate of deep-sea sediments (millimetre per thousand years) results in a better time resolution but requires much larger sample volumes. With regard to other potential ^244^Pu sources, we note that natural ^244^Pu production on Earth is negligible and the ESS abundance has decayed to 10^−17^ of its pre-solar value^22^,^23^. Anthropogenic production from atmospheric nuclear bomb tests and from high-power reactors is restricted to the last few decades, localized in upper layers and can easily be monitored through the characteristic isotopic fingerprint of the other co-produced ^239–242^Pu isotopes. In fact the detection of anthropogenic ^239,240^Pu in deep-sea sediments^38^,^39^,^40^ and crusts^41^ provides an excellent proxy for the ingestion efficiency of dust from the high atmosphere into these reservoirs, together with their chemical processing towards the final analyzed samples (Methods).

### Selected terrestrial archives for extraterrestrial ^244^Pu

Terrestrial archives like deep-sea FeMn crust and sediment archives extend over the past tens of million years. Large dust grains entering Earth’s atmosphere have also been observed by radar detections^42^. Extraterrestrial dust particles, cosmogenic nuclides and terrestrial input sink to the ocean floor and are eventually incorporated into the FeMn crust or sediment. For actinide transport through the latter stages, the observed deposition of global fallout^41^ from atmospheric nuclear bomb testing^38^,^39^ in deep marine reservoirs after injection to the stratosphere serves as a proxy to extraterrestrial particles.

**Figure 1:**
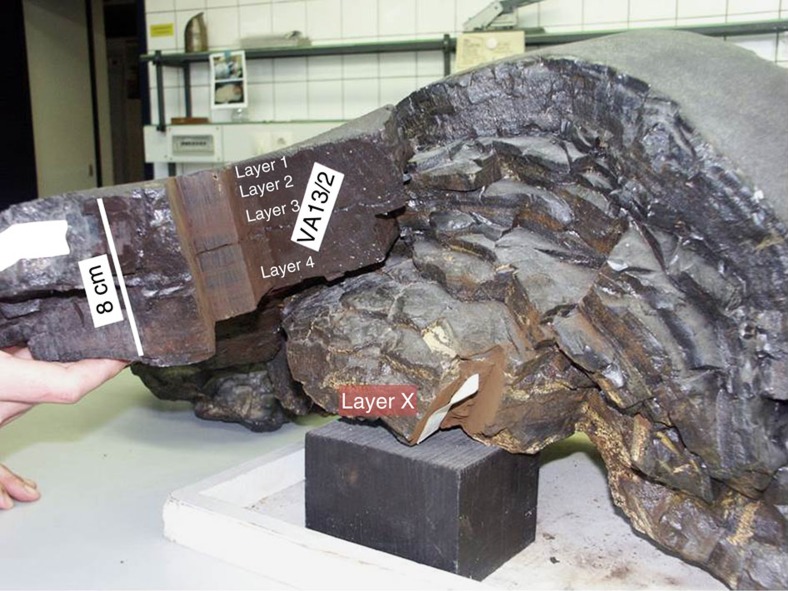
Crust sample 237 KD. This FeMn crust (with a total thickness of 25 cm) was sampled in 1976 from the Pacific Ocean at 4,830 m water depth: large samples used in this work were taken from one part of the crust (hydrogenous crust, layers 1–4, left in the figure)) and from the bottom (hydrothermal origin, layer X, crust started to grow ~65 My ago^43^, see also refs 44, 45).

We chose two independent archives: a large piece (1.9 and 0.4 kg samples) from a deep-sea manganese crust (237 KD from cruise VA13/2, collected in 1976) with a growth rate between 2.5 mm per My (refs 29, 37) and 3.57 mm per My (ref. 43). It originates from the equatorial Pacific (location 9°180′N, 146°030′W) at a depth of 4,830 m and covers the last ~25 My (refs 30, 43, 44, 45). In the very same crust, the live ^60^Fe signal mentioned above was found at about 2.2 My before present (BP)^28^,^29^. Our second sample, also from the Pacific Ocean, is a piston-core deep-sea sediment (7P), extracted during the TRIPOD expedition as part of the Deep-Sea Drilling Project (DSDP) at location 17°30′ N, 113°00′ W at 3,763 m water depth and covers a time period of ~0.5–2.1 My BP (W. Smith, Scripps Geological Collections, USA, personal communication). The crust sample, covering a total area of 227.5 cm^2^ and a time range of 25 My, was split into four layers (1–4) representing different time periods in the past (see Table 1). Each layer was subdivided into three vertical sections (B, C and D) with areas between 70 and 85 cm^2^, totalling 12 individually processed samples. The surface layer (layer 1, with a time range from present to 500,000 years BP) contains also the anthropogenic Pu signal originating from global fallout of atmospheric weapons testing^38^,^39^. Next, layer 2 spans a time period from 0.5–5 My BP, layer 3 5–12 My and layer 4 12–25 My (ref. 30). We note, the age for samples older than 14 My, where no ^10^Be dating is possible^29^,^37^, is more difficult to establish; different age models suggest a time period of 12 to ~18–20 My (ref. 44), another model up to ~30 My (ref. 45) for layer 4). Finally, sample X, the bottom layer of hydrothermal origin (Fig. 1) served as background sample.

For archives accumulating millions of years, the expected ^244^Pu abundance range (see discussion) is well within reach of accelerator mass spectrometry (AMS), an ultra-sensitive method^46^,^47^,^48^ of ion identification and detection. Based on the ingestion efficiency of Pu into deep-sea manganese crusts (21%) and on the AMS ^244^Pu-detection efficiency (1 × 10^−4^, see Methods), we calculate a measurement sensitivity expressed as a ^244^Pu flux onto Earth of the order of 0.1 to 1 atom per cm^2^ per My ^244^Pu from ISM deposition. Thus, for the crust with a 25 My accumulation period and with 200 cm^2^ surface area ~500–4,000 ^244^Pu-detection events are expected, and about a factor 100 less for the sediment sample (1.64 My time period and 4.9 cm^2^ surface area).[Table t2]

### AMS experimental data of ^244^Pu abundances in Earth archives

[Table t3]We have developed the capability to detect trace amounts of ^244^Pu in terrestrial archives by AMS^46^ and our technique provides background-free ^244^Pu detection with an overall efficiency (atoms detected/atoms in the sample) of ~1 × 10^−4^ (see Methods and Supplementary Tables 1–4). The AMS measurements determine the atom ratio ^244^Pu/^A^Pu where ^A^Pu (A=236 or 242) is a spike of known amount (added during the chemical processing of the sample) from which the number of ^244^Pu nuclei in the sample is obtained (see Methods). In addition to ^244^Pu counting, we also measured the shorter lived ^239^Pu (*t*_1/2_=24.1 ky) content as an indicator of anthropogenic Pu signature. [Table t2]

**Figure 2:**
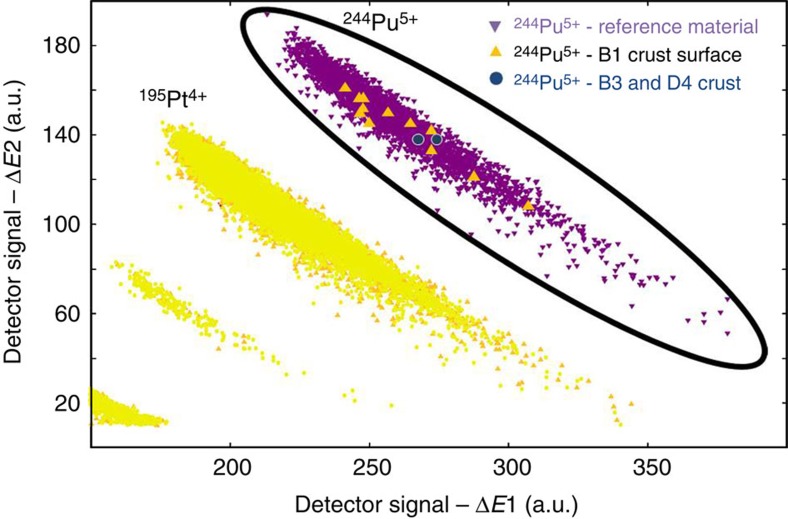
^**244**^ **Pu detection with AMS.** Identification spectra obtained in the AMS measurements with a particle detector (two independent differential energy-loss signals (Δ*E*1 and Δ*E*2) are plotted in *x*- and *y*-axis). Parasitic (or background) particles of different energy (for example, ^195^Pt^4+^) and different mass were clearly separated and do not interfere. Displayed is an overlay of ^244^Pu^5+^ events obtained in a series of measurements for a ^244^Pu reference material (purple triangle), the 12 events registered for one of the surface layer samples, B1 (yellow triangle) and the 2 events measured for the deeper layers B3 and D4, respectively (blue circle).

The results for the four crust layers and the blank sample, obtained from the AMS measurements on 11 individual crust samples, are listed in Tables 1 and 3 (see also Supplementary Tables 1 and 2; identification spectra are plotted in Fig. 2). We observed one single event in each of the two crust subsamples, namely layer 3, section B (B3), and layer 4, section D (D4). No ^244^Pu was registered in the other seven crust subsamples or the blank sample (X). A clear anthropogenic ^239^Pu and ^244^Pu signal, originating from atmospheric atomic-bomb tests from ~1950 to 1963, was observed in the top layer (16 events of ^244^Pu detected). Measurements of samples from deep layers (>0.5 My) show also some events during the ^239^Pu measurement (compared with the top layer, the ^239^Pu count rate in the deep crust layers were a factor of ~100 lower, and the one in the sediment and blank sample were a factor of ~1,000 lower). Since naturally produced ^239^Pu in these older layers is considered negligible, its presence is attributed to ^238^U still present in the final AMS sample at about 8 to 9 orders of magnitude higher than ^239^Pu and mimicking ^239^Pu detector events (see Methods); we also note that the ^236^Pu spike added for tracing the measurement efficiency was found to contain some ^239^Pu, which we corrected for, see Supplementary Table 4). We conclude from the ^239^Pu data that anthropogenic contamination did not add any significant ^244^Pu detector events for all older layers (using the anthropogenic ^244^Pu/^239^Pu ratio obtained from the top layer, 1 × 10^−4^, see Methods, Table 3). In the following, we calculate for all cases 2*σ* upper limits, that is, 95% confidence levels for which 0 (1 or 2) ^244^Pu events corresponds to an upper limit of <3 (5 or 6.7, respectively) ^244^Pu events (applying statistics for small signals^49^).

### ^244^Pu flux deduced from measured terrestrial concentrations

The crust data for all sections and for all three deeper layers are compatible (for details see also Supplementary Table 1). Owing to a higher chemical yield (integral sensitivity, column 5, Table 1) layers 3 and 4 provide lower limits. The measured ^244^Pu concentration in these layers can be converted into a ^244^Pu particle flux using chemical yield, detection efficiency, the incorporation efficiency of Pu into the crust (21±5 %, see Methods and Table 3), and the area and time period covered. We also assume that the extraterrestrial ^244^Pu flux through Earth’s cross-section is homogeneously distributed over the Earth’s surface. Hence, the interstellar flux is calculated by multiplying the measured flux into the crust by a factor of 4/0.21=19. We thus derive a 2σ limit^49^ for the ^244^Pu-ISM-flux at Earth orbit from data of the three layers <3,500, <1,300 and <1,560 ^244^Pu atoms per cm^2^ per My, and the one ^244^Pu event in layers 3 and 4 corresponds to a flux of 247^+1,000^_−235_ and 320^+1,250^_−300_ atoms cm^−2^ per My, respectively. Combining all samples (2 ^244^Pu events) a flux of 250^+590^_−205_ and a 2σ-limit on the ^244^Pu flux of <840 ^244^Pu atoms per cm^2^ per My is obtained. The data are plotted in Fig. 2. The single ^244^Pu event measured for the deep-sea sediment converts to a flux of 3,000 (<15,000) atoms per cm^2^ per My. Both archives give consistent ^244^Pu flux limits with a higher sensitivity for the crust samples.

## Discussion

First, we estimate the expected ^244^Pu flux from ISM dust particles penetrating the SS, and their incorporation into terrestrial archives. Our experimental results are then compared with these estimations. Based on a uniform production model^18^ or an open-box model 5,16,50 (see also ref. 24), taking into account Galactic-disk enrichment in low-metallicity gas, the present-day ISM atom ratio ^244^Pu/^238^U from SN events is calculated to be between 0.017 and 0.044 (Table 2). We further assume that the abundance of ^238^U in ISM dust is the same as that of chondrite meteorites^51^ (corrected for the SS age), 1.7 × 10^−8^ g per g meteorite, and derive a steady-state ^244^Pu abundance of (2.8–7.5) × 10^–10^ g Pu per g ISM (that is, (0.8–2.2) × 10^−14^ atoms per cm^3^); similar values are obtained if normalized to ^232^Th.

For interstellar dust particles (ISDs) entering the SS^27^, we have to take into account filtering when penetrating the heliosphere. ISDs were observed by the Ulysses, Galileo and Cassini^31^,^52^ space missions over more than 5 years, for distances from the Sun between 0.4 and >5 AU. Measurements of the Cassini space mission^27^,^31^ determine a mean flux of ISM dust of (3–4) × 10^−5^ particles per m^2^ per s at a distance of 1 AU, that is, at Earth’s position, with a mean particle mass of (3–7) × 10^−13^ g (0.5–0.6 μm average particle size). These particles show a speed distribution corresponding to the flow velocity of the ISM (26 km s^−1^) and constitute 3–9% of the dust component of the ISM intercepted by the SS (see Table 2). The direct collection of a few particles identified as ISD, very recently reported^53^, although of low statistical significance, supports the scenario of penetration of large ISD particles into the inner SS and may be consistent with the satellite data. It should be noted that Galactic cosmic-rays penetrate the SS and recent observations clearly demonstrate therein the presence of Th and U, and tentatively of ^244^Pu (ref. 54).

Within the assumptions described above, the expected flux of ^244^Pu atoms from the ISM reaching the inner SS (at Earth orbit) is (2.5–21) × 10^−31^ g Pu per cm^2^ per My or 20,000–160,000 ^244^Pu atoms per cm^2^ per My. If evenly distributed over the Earth’s surface (that is, assuming a unidirectional ISM flux) the ^244^Pu flux into terrestrial archives becomes 5,000–40,000 ^244^Pu atoms per cm^2^ per My.

**Figure 3:**
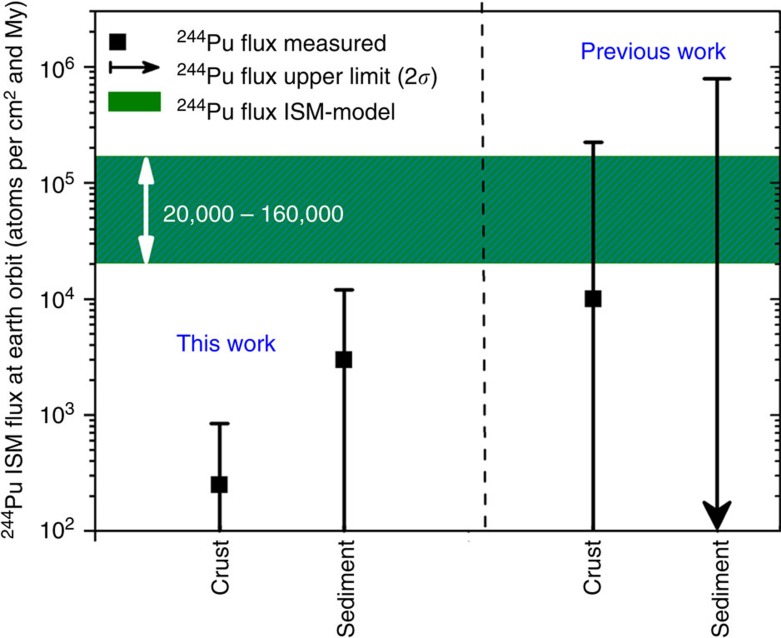
Comparison of the measured ^244^Pu flux at Earth orbit with models. The ISM ^244^Pu flux at Earth orbit was determined from the concentrations measured in a deep-sea crust and a deep-sea sediment sample (note the logarithmic scale). Our results are compared with previous measurements (deep-sea crust^41^ and sediment^40^) and to models of galactic chemical evolution^18^,^50^ assuming steady-state conditions and taking into account filtration of dust particles when entering the heliosphere^31^. The arrows and error bars represent upper levels (2*σ*, 95% confidence levels) from the measurements. The green area indicates the data range deduced from the steady-state models. The crust data suggest a ^244^Pu flux, which is a factor between 80 and 640 lower than inferred from the models.

Our experimental results (Table 1) provide for the first time a sensitive limit of interstellar ^244^Pu concentrations reaching Earth, integrated over a period of 24.5 My. Our data are a factor of 80–640 lower than the values expected under our constraints on ISM grain composition from a SN derived steady-state actinide production (the 2*σ* upper limit of ~840 atoms per cm^2^ per My is still a factor of 25–200 lower). The lifetime of ^244^Pu is comparable to the complete mixing time scales of the ISM^32^,^33^,^34^. The deep-sea crust sample integrates a ^244^Pu flux over a time period of 24.5 My (~1/10 of the SS rotation period in the Galaxy) corresponding to a relative travel distance of the SS of 650 pc (taking the mean speed of the measured ISM dust particles of 26 km s^−1^ (ref. 52), ~1/10 of the galactic orbital speed, as a proxy for ISM reshaping and for motion differences relative to the co-rotating local neighbourhood). These results, consistent with previous studies on extraterrestrial ^244^Pu in crust^41^,^55^ and sediment samples^40^,^56^, are more sensitive by a factor of >100 and provide for the first time stringent experimental constraints on actinide nucleosynthesis in the last few hundred million years (see Fig. 3).

A simple steady-state scenario might represent a simplified assumption within our local ISM environment. Compared with the typical size of ISM substructures of ~50–100 pc (for example, LB)^27^,^32^,^33^,^34^,^57^ and life-times of some 10 My, the crust sample probes, however, the equivalent of ~10 such cavities (the ^244^Pu life-time coupled with the spatial movement of the SS during the 24.5 My accumulation). Thus, we expect existing ISM inhomogeneities largely smeared out in our space- and time-integrated samples, confirming the significance of a ratio <1/100 between measured and expected ^244^Pu abundance.

Further, we can relate our result to actinide nucleosynthesis during the recent SN history of the LB^32^,^33^,^34^,^58^,^59^ in which the SS is embedded now. ISM simulations suggest the LB was formed by ~14–20 SN explosions within the last 14 My (refs 32, 33, 34) with the last one ~0.5 My BP (refs 32, 58). To reproduce size and age of the LB, an intermediate density of seven particles per cm^3^ (~10 times the mean density of the local environment now) before the first SN explosion took place, is required^34^. The mean mass density of the LB has since transformed to 0.005 particles per cm^3^. The series of SNe explosions has generated the void inside the LB and has continuously pushed material into space forming an ISM shell. The SS is now placed inside the LB and thus has passed or passes the front of accumulated swept-up material including possible pre-existing ^244^Pu from nucleosynthesis events prior to the formation of the LB^35^,^36^.

We can distinguish three different scenarios for the recent LB history: (i) the SN activity transformed the local ISM from a dense to a low-density medium (LB), and pre-existing ISM material containing (steady state) old ^244^Pu was swept-up and passed the SS^35^,^60^; (ii) direct production of ^244^Pu in the 14–20 SNe and their expected traces left on Earth^35^; and (iii) independently, we can compare our data for ^244^Pu with recent AMS data of ^60^Fe influx^28^,^29^.

In a simple first order estimate for scenario (i), we assume that the swept-up material is distributed over a surface with a radius of 75 pc. Using the pre-LB density of seven particles per cm^3^ (ref. 34) with 1% of this ISM mass locked into dust, we calculate with our assumptions of Pu concentration in dust (Table 2) and a dust penetration efficiency of ~6±3% into the SS to Earth orbit, a ^244^Pu fluence from swept-up material of (0.4–3) × 10^6 244^Pu atoms per cm^2^ (see Table 2).

Our experimental data give a flux of 200^+800^_−200_^244^Pu atoms per cm^2^ per My for the last 12 My (layers 2 and 3) at Earth orbit corresponding to a fluence of 2,300 (<12,000) ^244^Pu atoms per cm^2^ during this period. This experimental value for the fluence is a factor of ~170–1,300 lower than the value calculated above (see Table 2) assuming swept-up material of about half the diameter of the LB is moved across the SS. We deduce approximately the same discrepancy as found for a simple steady-state actinide production scenario.

For LB scenario (ii), in a first order estimation, we take the SN-rate of 1.1–1.7 SNe/My within the LB^34^,^58^ and a mean distance to the SS for these SN events of 100 pc. From our measured value of 200^+800^_−200_^244^Pu atoms per cm^2^ per My at Earth orbit (with 6% penetration efficiency into 1 AU), this corresponds to ~3,000 (<17,000) ^244^Pu atoms per cm^2^ per My unfiltered ISM flux, spread over a surface area with a radius of 100 pc. We deduce an average ^244^Pu yield per SN of (0.6^+2.4^_−0.6_) × 10^−9^ M_solar_ for the last 12 My.

Finally for LB scenario (iii), Knie *et al*.^28^ and Fitoussi *et al*.^29^ measured a clear ^60^Fe signal of possible SN origin ~2.2 My in the past in exactly the same crust material (237 KD) as we have used in this work for the search of ^244^Pu (using a sample ~50 cm distant; a SN origin for ^60^Fe is being questioned by some authors^61^,^62^, while several recent studies on ^60^Fe in deep ocean sediments^63^,^64^ and in lunar samples^65^ confirm the results of Knie *et al*.^28^). Thus we can directly compare the measured fluences of ^60^Fe and ^244^Pu for the same event (using layer 2, 0.5–5 My). These fluence values can be converted into an atom ratio that is independent of the SS penetration efficiency and we assume the same incorporation efficiency for Fe and Pu (refs 63, 64, 65, 66). We deduce a ^244^Pu/ ^60^Fe isotope ratio for this event of <6 × 10^−5^ (similarly, we obtain an upper limit from the sediment of <10^−4^). Clearly, this ratio depends strongly on the type of explosive scenario. Literature values for this ratio are highly varying also due to large uncertainties in the *r*-process yields.

Our experimental results indicate that SNe, at their standard rate of ~1 to 2 per 100 years in the Galaxy, did not contribute significantly to actinide nucleosynthesis for the past few hundred million years and actinide nucleosynthesis, as mapped through live ^244^Pu, seems to be very rare. Our data may be consistent with a predominant contribution of compact-object mergers, which are 10^2^ to 10^3^ less frequent than core-collapse SNe^1^. A recent observation indicates indeed that such mergers may be sites of significant production of heavy *r*-process elements^10^,^11^. Our experimental work is also in line with observations of low-metallicity stars^12^,^14^, indicating splitting into a rare and a more frequent *r*-process scenario allowing an independent evolution of the *r*-process elements Eu/Th over time^3^,^4^. In addition, we must conclude from our findings that, given the presence of short-lived actinide ^244^Pu (and ^247^Cm) in the ESS, it must have been subject to a rare heavy *r*-process nucleosynthesis event shortly before formation.

## Methods

### Details on the chemistry of the crust samples

Quantitative extraction of Pu was required from the 2.3 kg crust sample. The sample potentially contained some 10^6^–10^7^ atoms of ^244^Pu, which correspond to an atom-concentration of (0.5–5) × 10^−19^ relative to the bulk material. No stable isobar to ^244^Pu exists in nature and molecular interference in the measurements is excluded. The FeMn crust sample was split into four layers 1–4 and three sections B, C and D. The top layer (1 mm, ‘crust modern’) was removed for measuring the anthropogenic Pu content originating from atmospheric atomic-bomb tests from ~1950 to 1963. Four different vertical layers represent different time periods in the past, while three different horizontal sections were chosen to identify possible lateral variations (B, C and D). The 12 individual pieces had masses between 30 and 360 g. Ten samples were measured by AMS.

The individual parts of the crust were dissolved in aqua regia and H_2_O_2_, and a spike of ~3 × 10^8^ atoms of a ^236^Pu reference material was added to the leached solutions. After removal of the undissolved SiO_2_ fractions, the solutions were brought to dryness and successively redissolved in concentrated HNO_3_ and H_2_O_2_. At this stage, the sample solutions contain the actinides, but also the dominant fraction of the matrix elements of the crust, in particular Mn (~14 to 28%) and Fe (~16 to 28%). To separate the actinides from the Mn and Fe fraction, a pre-concentration step involving the selective co-precipitation of the actinides with CaC_2_O_4_ at pH ~1.7 was performed. After centrifugation, the precipitated CaC_2_O_4_ was converted to CaCO_3_ in a muffle furnace at a temperature of 450 °C for several days. The CaCO_3_ was dissolved in 7.2 M HNO_3_ and the oxidation state of Pu was adjusted quantitatively to (IV)Pu by the addition of NaNO_2_. These solutions were then loaded onto pre-conditioned anion-exchange columns containing Dowex 1 × 8, from which after the separation of the Ca, Am, Cm and the Th fractions Pu was eluted by reduction to (III)Pu with a solution of HI. To purify the obtained Pu fraction, two additional successive anion-exchange separations similar to the one described above were performed on the eluted Pu solutions. The Pu fractions were further purified from the organic residues by fuming with HNO_3_ and H_2_O_2_. Successively an Fe(OH)_3_ co-precipitation of Pu was performed in 1 M HCl by adding 2 mg of Fe powder. After centrifugation and drying of the precipitate, the Fe(OH)_3_ was converted to iron oxide by combustion in a muffle furnace at 800 °C for 4 h. The plutonium oxide embedded in a matrix of iron oxide was then mixed with 2 mg of high-purity Ag powder and pressed in the sample holders suitable for the subsequent AMS measurement.

Owing to the massive matrix component of the crust for some samples a low chemical yield was observed (in the AMS measurements via the ^236^Pu spike). In these cases the procedure for Pu extraction was repeated, that is, the solutions containing the Mn and Fe fraction underwent again a CaC_2_O_4_ co-precipitation procedure and the resulting actinide fractions were mixed with the rest of the remaining fractions originating from the first threefold anion-exchange separation. These solutions underwent a chromatographic column separation employing Tru-resin in 5 M HCl, from which after the elution of the Ca, Am and Cm and the Th fractions Pu was stripped out with a solution of 0.03 M H_2_C_2_O_4_ in 0.5 M HCl.

### Chemical processing of the TRIP deep-sea sediment

Similarly, Pu was extracted from two deep-sea sediment samples of 43 and 58 g mass provided by the Scripps Oceanographic Institute, the University of California at San Diego. It was a piston core (7P), extracted during the TRIPOD expedition (1966) as part of the DSDP at location 17°30′ N 113°00′ W (Pacific Ocean) at 3,763 m water depth. Two main sections were sampled with sediment depths 0–80 cm and 80–230 cm, from which the top 3 cm (containing the anthropogenic Pu) were removed. One quarter of the total cross-section throughout the ~230 cm length of the core was used in this study (4.9 cm^2^). The samples, shipped in sealed polyethylene, were chemically processed at the Hebrew University, Jerusalem.

The physical and chemical processing of the sediments is as follows: the processing involved brief milling and calcination of the sample, alkali fusion of the sediment using NaOH at 750 °C, liquid-phase extraction of Fe and other main elements and liquid ion-chromatography to extract the Pu fraction. Prior to the alkali fusion steps, an isotope ^242^Pu marker and a chemical ^230^Th marker were added to the sediment. ^242^Pu was used to monitor the efficiency of Pu detection by measuring the ^242^Pu content of the final AMS sample (analogous to the ^236^Pu spike in the crust samples), while ^230^Th served as additional indicator of the chemical efficiency of actinide extraction by measuring the alpha activity of an electroplated deposition prepared from a separate fraction. The final AMS samples were obtained by co-precipitation of the Pu fraction with Fe in an ammonia solution, centrifugation and ignition to obtain a Fe_2_O_3_ matrix containing the Pu marker and traces. Finally, 2 mg of high-purity Ag powder was added and the powder pressed in the sample holders suitable for the subsequent AMS measurement.

### AMS-measurement procedure

We have applied the most sensitive technique—AMS^46^,^47^,^48^ to detect minute amounts of ^244^Pu. This technique provides the complete suppression of any interfering background, for example, molecules of the same mass, by the stripping process in the terminal of a tandem accelerator, which is crucial for such experiments where only a few counts are expected. The ^244^Pu measurements were performed at the Vienna Environmental Research Accelerator (VERA) facility in the University of Vienna^46^,^67^,^68^,^69^. This set up has been optimized for high-measurement efficiency and offered an exceptional selectivity.

The individual crust samples were spiked with a well-known amount of ^236^Pu atoms (^242^Pu for the sediment). ^244^Pu and ^239^Pu measurements were performed relative to the ^236^Pu (^242^Pu) spike, that is, the total efficiency and the chemical yield of Pu (when compared with the theoretical measurement efficiency) were monitored with the ^236^Pu (^242^Pu) spike, that was counted in short time intervals before and after the ^244^Pu runs in the AMS measurements. The chemical yield varied between 5 and 70% largely depending on the sample matrix.

In a sputter source, the Fe/Ag matrix, containing the Pu atoms, was bombarded with Cs ions and negative PuO ions were extracted, and energy and mass were analyzed before injection into the tandem accelerator. The negative ions were accelerated to the 3 MV tandem terminal and stripped there to positive ions in the gas stripper (O_2_). ^244^Pu^5+^ ions were accelerated to a final energy of 18 MeV and selected with a second analyzing magnet. They then had to pass an additional energy and another mass filter (electrostatic and magnetic dipoles, respectively) and were finally counted in an energy-sensitive particle detector. The system was optimized with a ^238^U pilot beam and monitored during a measurement with reference samples containing ^242^Pu. The measurement set up for ^244^Pu and ^236^Pu counting was scaled from the tuning set up. At the end of a measurement series, reference samples containing a well-known isotope ratio of ^244^Pu/^242^Pu were measured. The measured ratios reproduced the nominal values within 4% and confirmed the validity of scaling between the different masses in the measurement.

This set up suppresses adjacent masses (for example, ^238^U from ^239^Pu) by 8–9 orders of magnitude. This suppression factor was sufficient for ^244^Pu counting as no interference from neighbouring masses is expected for ^244^Pu (and additional isotopic suppression, for example, via time-of-flight identification, would have been at the cost of lower particle transmission). However, ^238^U was abundant at levels of ~10 p.p.m. in the crust, and U separation from Pu in the chemical preparation of these samples was not 100%. Thus, the detector events registered for ^239^Pu counting, by 2–3 orders of magnitude lower compared with modern samples, are attributed to leaking ^238^U atoms injected as ^238^UOH^−^ ions together with ^239^PuO^−^ mimicking ^239^Pu. To summarize, our detector event rate for ^239^Pu suggests no significant anthropogenic ^244^Pu contamination.

The measurement procedure was a sequence of alternating counting periods of ^236^Pu, ^239^Pu, ^244^Pu and again ^236^Pu. All samples were repeatedly measured until they were completely exhausted. The sputtering time per sample was between 5 and 20 h. Three measurement series were required to fully consume all the samples. The overall yields for the 12 crust samples were between 0.06 × 10^−4^ and 1.54 × 10^−4^, that is, one ^244^Pu detector event would represent correspondingly between 6,500 and 1.7 × 10^5 244^Pu atoms in the analyzed sample. A similar procedure was followed for the sediment samples where ^242^Pu was used as a spike.

Due to the low number of expected detector events, the machine and measurement backgrounds were carefully monitored with samples of pure Fe and Ag powders. They were sputtered identical to the samples containing the crust fractions. In addition, one crust sample (X), the lowest layer of the crust material, was of hydrothermal origin where no extraterrestrial ^244^Pu could accumulate. This sample was chemically prepared and measured in the same way as the other crust samples and served as a process blank for potential chemistry and machine background.

### Incorporation efficiency of Pu into the deep-sea crust

The incorporation efficiency of bomb-produced Pu into the crust was determined from the anthropogenic ^239^Pu content in the top layer of the crust and deep-sea sediment data (details are given in Table 3). The total measurement efficiency is calculated from the total number of ^236^Pu registered and normalized by the time fraction of ^236^Pu AMS counting and divided by the number of ^236^Pu atoms added as spike to the sample (3 × 10^8^ atoms each). The number of ^244^Pu atoms per sample is calculated from the number of ^244^Pu events registered with the particle detector, scaled by the time fraction of AMS ^244^Pu counting time and normalized with the measurement efficiency; the same procedure was used for ^239^Pu atoms per sample. The ^239^Pu detector events were corrected for a well-known contribution when adding the ^236^Pu spike, which also contains ^239^Pu (see Supplementary Table 1 for more details).

The average over 18 sediment cores from the Pacific measured between 1974 and 1979 gave a ^239,240^Pu sediment inventory of 2.15 Bq m^−2^ (ref. 38). When compared with the well-known surface activity of 2.8 mCi km^−2^ (104 Bq m^−2^)^39^, at the time of sampling the crust in 1976, 2.1% of anthropogenic Pu from the bomb tests was incorporated into deep-sea sediments with sediments having an incorporation efficiency of 100% (this compares well with the ratio of the time that had passed since the peak in atmospheric bomb-testing (15 years) and the mean residence time of Pu in the ocean of ~440 years; this ratio is, 3.4%). Taking the total anthropogenic Pu-inventory at the location of the crust (between 60 and 78 Bq m^−2^ and a ^240^Pu/^239^Pu atom ratio of 0.20; refs 40, 41); and the fraction of 2.1% measured in sediments, we found that 8.2 × 10^7 239^Pu atoms per cm^2^ have reached the crust in the year 1976. We measured from the three crust subsamples from the top layer a ^239^Pu surface density of (1.76±0.44) × 10^7^ cm^−2^, and thus deduce an incorporation efficiency into the crust of (21±5)%. We assume that the ISM-Pu is incorporated like the bomb-produced Pu.

## Author contributions

A.W. performed the data analysis and wrote the main paper together with M.P., and all authors discussed the results and commented on the manuscript. K.K., T.F. and G.K. organized the crust sample; M.P. provided the sediment sample. K.K. and F.Q. were primarily responsible for sample preparation of the crust; A.O and C.F. for the preparation of the sediment. P.S., A.W. and K.K. performed the AMS measurements.

## Additional information

**How to cite this article:** Wallner, A. *et al*. Abundance of live ^244^Pu in deep-sea reservoirs on Earth points to rarity of actinide nucleosynthesis. *Nat. Commun.* 6:5956 doi: 10.1038/ncomms6956 (2015).

## Supplementary Material

Supplementary Information Supplementary Figure 1, Supplementary Tables 1-4 and Supplementary References.

## Figures and Tables

**Table 1:** ^244^Pu detector events and corresponding ISM flux compared with galactic chemical models assuming steady state.

**Deep-sea archive**	**Time period (My)**	**Sample area (cm** ^**2**^ **)**	**Sample mass (g)**	**Integral sensitivity (eff. × area × time period) (cm** ^**2**^ **My)**	^**244**^ **Pu detector events (2*σ* limit)** *	^**244**^ **Pu flux into terrestrial archi** **ve (atoms per cm** ^**2**^ **per My)**	^**244**^ **Pu flux ISM at Earth orbit (atoms per cm** ^**2**^ **per My)** †
Crust_modern	0–0.5	227.2	80	0.006	16	—	—
Layer X	Blank	~100	364	—	0	—	**—**
Layer 2	0.5–5	227.2	473	0.016	0 (<3)	**<188**	**<3,500**
Layer 3	5–12	227.2	822	0.075	1 (<5)	**13** ^**+53**^_**−12**_ **(<66)**	**247** ^**+1,000**^_**−235**_
Layer 4	12–25	142.2	614	0.060	1 (<5)	**17** ^**+66**^_**−16**_ **(<83)**	**320** ^**+1,250**^_**−300**_
**Crust**	**0.5–25**	**182**	**1,909**	**0.151**	**2 (<6.7)**	**13** ^**+31**^_**−11**_ **(<44)**	**250** ^**+590**^_**−205**_
**Sediment**	**0.53–2.17**	**4.9**	**101**	**0.0013**	**1 (<5)**	**750** ^**+3,000**^_**−710**_	**3,000** ^**+12,000**^_**−2,850**_
**Model and satellite data** ‡		**Steady-state model and ISM flux data at 1** **AU from satellite Cassini**					**20,000–160,000**

eff., efficiency; ISM, interstellar medium.

The FeMn crust sample was split into four layers 1–4 (see Methods). The top layer (1 mm, ‘crust modern’) was removed for measuring the anthropogenic Pu content. In total two ^244^Pu detector events were registered using AMS in all older crust samples over a 72 h counting time (column 6). We calculate from our data an extraterrestrial ^244^Pu flux and a 2*σ* limit from<6.7 extraterrestrial ^244^Pu events^49^. The sediment sample also gave one ^244^Pu detector event and none were registered in any of the blank samples. The term ‘integral sensitivity’ represents a quantity that combines the overall measurement eff., the flux integration area and the time period covered by the individual samples.

^*^Because of the low ^244^Pu event rate, we also display 2σ upper levels (95% confidence levels) applying low-level statistics^49^.

^†^Using an incorporation efficiency *ε*=(21±5)% for the crust and 100% for the sediment sample (Methods). The mean area for the crust sample is 182 cm^2^ (accounting for the different time periods) and 4.9 cm^2^ for the sediment sample. For calculating the ISM flux at Earth orbit, the measured ^244^Pu flux into the terrestrial archives was corrected for the incorporation efficiency and was multiplied by a factor of 4 to account for the ratio of Earth’s surface to its cross-section (that is, assuming a unidirectional and homogeneous ISM flux relative to the Solar System).

^‡^the steady-state ^244^Pu flux is based on the actinide (U and Th) abundances measured in meteorites, and on present-day Pu/U and Pu/Th ISM concentrations deduced from galactic chemical evolution models. The Pu flux at 1 AU (Earth orbit) is corrected for the filtration of interstellar dust particles when entering the heliosphere of our Solar System (3–9%, see Methods).

**Table 2 t2:** Expected ^244^Pu fluxes at 1 AU from models and satellite data.

**Table 3 t3:** Anthropogenic Pu at the surface and the incorporation efficiency into the manganese crust.

	**Surface layer 1**				**Blank**
Time period	0–0.5 My				—
	Contains anthropogenic Pu (top 1 mm)				Hydrothermal
					~100 cm^2^
Subsample	B1	C1	D1	Total	X
Mass (g)	32	20	28	80	364
Time period (My)	0–0.5	0–0.5	0–0.5	0–0.5	—
Total meas. eff. (10^−4^)	0.82	0.45	0.18	<0.51>	0.93
Measuring time	3.8 h	3.8 h	2.6 h	10.2 h	3.4 h
^236^Pu atoms spike	3 × 10^8^	3 × 10^8^	3 × 10^8^	9 × 10^8^	3 × 10^8^
^244^Pu atoms (10^4^)	18.5	5.3	13.3	37.1	**<1.7**
^239^Pu atoms (10^8^)	13.9	15.6	9.9	39.4	**0.3**
^**244**^ **Pu/** ^**239**^ **Pu (10** ^**−4**^ **)**	1.3	0.3	1.3	1.0±0.3	**—**
^239^Pu atoms per cm^2^ measured	1.6 × 10^7^	2.2 × 10^7^	1.4 × 10^7^	1.76 × 10^7^	—
^239^Pu atoms per cm^2^ reaching deep-sea floor* in 1976 (refs 38, 39)	—	—	—	8.2 × 10^7^	—
^**239**^ **Pu incorporation eff. (crust)**	—	—	—	(21±5)%	

eff., efficiency; meas., measurement.

237 KD (VA13/2) deep-sea crust measurement: detailed data for the surface layer 1 (anthropogenic Pu) and the hydrothermal blank sample and determination of the Pu incorporation eff. into the deep-sea manganese crust by comparison of the known amount of atomic bomb-produced Pu at the crust’s location with the measured Pu in the top layer 1.

^*^The amount of ^239^Pu atoms per cm^2^ reaching the deep-sea floor at the time of crust sampling (1976) is derived from the ratio (2.1 %) of the ^239,240^Pu fluence measured in deep-sea sediments^39^ (assumed to incorporate 100% of precipitated material) and the overall ^239,240^Pu fallout fluence measured for the location of the crust^41^.

## References

[b1] Qian Y.-Z. The origin of the heavy elements: recent progress in the understanding of the *r*-process. Prog. Part. Nucl. Phys. 50, 153–199 (2003).

[b2] Woosley S. E. & Weaver T. A. The evolution and explosion of massive stars. II. Explosive hydrodynamics and nucleosynthesis. Astrophys. J. Suppl. Ser. 101, 181–235 (1995).

[b3] Arnould M., Goriely S. & Takahashi K. The *r*-process of stellar nucleosynthesis: astrophysics and nuclear physics achievements and mysteries. Phys. Rep. 450, 97–213 (2007).

[b4] Thielemann F. K. . What are the astrophysical sites for the *r*-process and the production of heavy elements? Prog. Part. Nucl. Phys. 66, 346–353 (2011).

[b5] Meyer B. S. & Clayton D. D. Short-lived radioactivities and the birth of the sun. Space Sci. Rev. 92, 133–152 (2000).

[b6] Arcones A. & Martinez-Pinedo G. Dynamical *r*-process studies within the neutrino-driven wind scenario and its sensitivity. Phys. Rev. C. 83, 045809 (2011).

[b7] Goriely S. . New fission fragment distributions and *r*-process origin of the rare-Earth elements. Phys. Rev. Lett. 111, 25402 (2013). 10.1103/PhysRevLett.111.242502 24483647

[b8] Tanvir K. . A ‘kilonova’ associated with the short-duration γ-ray burst GRB130603B. Nature 500, 547–549 (2013). 23912055 10.1038/nature12505

[b9] Berger E., Fong W. & Chornock R. An *r*-process kilonova associated with the short-hard GRB 130603B. Astrophys. J. Lett. 774, L23 (2013).

[b10] Argast D., Samland M., Thielemann F.-K. & Qian Y.-Z. Neutron star mergers versus core-collapse supernovae as dominant *r*-process sites in the early Galaxy. Astron. Astrophys. 416, 997–1011 (2004).

[b11] Goriely S. & Arnould M. Actinides: how well do we know their stellar production? Astron. Astrophys. 379, 1113–1122 (2001).

[b12] Jacobson H. R. & Frebel A. Observational nuclear astrophysics: neutron-capture element abundances in old, metal-poor stars. J. Phys. G 41, 044001 (2014).

[b13] Thielemann F. . Heavy elements and age determinations. Space Sci. Rev. 100, 277–296 (2002).

[b14] Cowan J. J. & Sneden C. h. Heavy element synthesis in the oldest stars and the early Universe. Nature 440, 1151–1156 (2006). 16641987 10.1038/nature04807

[b15] Fields B. D., Truran J. W. & Cowan J. J. A simple model for *r* process scatter and halo evolution. Astrophys. J. 575, 845–854 (2002).

[b16] Huss G. R., Meyer B. S., Srinivasan G., Goswami J. N. & Sahijpal S. Stellar sources of the short-lived radionuclides in the early solar system. Geochim. Cosmochim. Acta 73, 4922–4945 (2009).

[b17] Diehl R. . Radioactive ^26^Al from massive stars in the Galaxy. Nature 439, 45–47 (2006). 16397491 10.1038/nature04364

[b18] Wasserburg G. J., Busso M., Gallino R. & Nollett K. M. Short-lived nuclei in the early Solar System: possible AGB sources. Nucl. Phys. A 777, 5–69 (2006).

[b19] Turner G., Harrison T. M., Holland G., Mojzsis S. J. & Gilmour J. Extinct ^244^Pu in ancient Zircons. Science 306, 89–91 (2004). 15459384 10.1126/science.1101014

[b20] Turner G. . Pu–Xe, U–Xe, U–Pb chronology and isotope systematics of ancient zircons from Western Australia. Earth Planet. Sci. Lett. 261, 491–499 (2007).

[b21] Kuroda P. K. Nuclear fission in the Early history of the Earth. Nature 187, 36–38 (1960).

[b22] Lachner J. . Attempt to detect primordial ^244^Pu on Earth. Phys. Rev. C85, 015801 (2012).

[b23] Hoffman D. C., Lawrence F. O., Mewherter J. L. & Rourke F. M. Detection of plutonium-244 in nature. Nature 234, 132–134 (1971).

[b24] Dauphas N. The U/Th production ratio and the age of the Milky Way from meteorites and Galactic halo stars. Nature 435, 1203–1205 (2005). 15988518 10.1038/nature03645

[b25] Hudson G. B., Kennedy B. M., Podosek F. A. & Hohenberg C. M. in *Proc. (A89-36486 15-91) Lunar and Planetary Science Conference, 19th*, Houston, TX, 14–18 March, 1988, 547–555 (Cambridge University Press/Lunar and Planetary Institute, Cambridge/Houston, TX, 1989).

[b26] Dwek E. The evolution of the elemental abundances in the gas and dust phases of the galaxy. Astrophys. J. 501, 643–665 (1998).

[b27] Mann I. Interstellar dust in the Solar System. Annu. Rev. Astron. Astrophys. 48, 173–203 (2010).

[b28] Knie K. . ^60^Fe anomaly in a deep-sea manganese crust and implications for a nearby supernova source. Phys. Rev. Lett. 93, 171103 (2004). 15525065 10.1103/PhysRevLett.93.171103

[b29] Fitoussi C. . Search for supernova-produced ^60^Fe in a marine sediment. Phys. Rev. Lett. 101, 121101 (2008). 18851357 10.1103/PhysRevLett.101.121101

[b30] Poutivtsev M. . Highly sensitive AMS measurements of ^53^Mn. Nucl. Instr. Meth. B 268, 756 (2010).

[b31] Altobelli N. . Interstellar dust flux measurements by the Galileo dust instrument between the orbits of Venus and Mars. J. Geophys. 110, A07102 (2005).

[b32] De Avillez M. A. & Low M.-M. M. Mixing Timescales in a Supernova-driven Interstellar Medium. Astrophys. J. 581, 1047–1060 (2002).

[b33] Fuchs B., Breitschwerdt D., de Avillez M. A., Dettbarn C. & Flynn C. The search for the origin of the Local Bubble redivivus. Mon. Not. R. Astron. Soc 373, 993 (2006).

[b34] Baumgartner V. & Breitschwerdt D. Superbubble evolution in disk galaxies: I. Study of blow-out by analytical models. Astron. Astrophys. 557, A140 (2014).

[b35] Ellis J., Fields B. D. & Schramm D. N. Geological isotope anomalies as signatures of nearby supernovae. Astrophys. J. 470, 1227 (1996).

[b36] Fields B. D., Hochmuth K. A. & Ellis J. Deep-ocean crusts as telescopes: using live radioisotopes to probe supernova nucleosynthesis. Astrophys. J. 621, 902–907 (2005).

[b37] Segl M. . ^10^Be-dating of a mangenese crust from the Central North Pacific and implications for ocean paleocirculation. Nature 309, 540–543 (1984).

[b38] Livingston H. D. & Anderson R. F. Large particle transport of plutonium and other fallout radionuclides to the deep ocean. Nature 303, 228–231 (1983).

[b39] Bowen V. T., Noshkin V. E., Livingston H. D. & Volchok H. L. Fallout radionuclides in the pacific ocean: vertical and horizontal distributions, largely from GEOSECS stations. Earth Planet. Sci. Lett. 49, 411–434 (1980).

[b40] Paul M. . Experimental limit to interstellar ^244^Pu abundance. Astrophys. J. Lett. 558, L133–L135 (2001).

[b41] Wallner C. . Supernova produced and anthropogenic ^244^Pu in deep sea manganese encrustations. N. Astron. Rev. 48, 145–150 (2004).

[b42] Baggaley W. J. Advanced meteor orbit radar observations of interstellar meteoroids. J. Geophys. Res. 105, 10353–10361 (2000).

[b43] Siebert C. h., Nägler T. F., von Blanckenburg F. & Kramers J. D. Molybdenum isotope records asa potential new proxy for paleoceanography. Earth Planet. Sci. Lett. 211, 159–171 (2003).

[b44] Frank M., O’Nions R. K., Hein J. R. & Banakar V. K. 60 Myr records of major elements and Pb–Nd isotopes from hydrogenous ferromanganese crusts: Reconstruction of seawater paleochemistry. Geochim. Cosmochim. Acta 63, 1689–1708 (1999).

[b45] Poutivtsev M. Extraterrestrisches ^53^Mn in hydrogenetischen Mangankrusten. PhD thesis, 2007 (Tech. Univ. Munich (2007).

[b46] Steier P. . AMS of the Minor Plutonium Isotopes. Nucl. Instr. Meth. B 294, 160–164 (2013). 10.1016/j.nimb.2012.06.017 PMC3617651 23565016

[b47] Synal H.-A. Developments in accelerator mass spectrometry. Int. J. Mass Spectrom. 349-350, 192–202 (2013).

[b48] W. Kutschera W. Applications of accelerator mass spectrometry. Int. J. Mass Spectrom. 349-350, 203–218 (2013).

[b49] Feldman G. J. & Cousins R. D. Unified approach to the classical statistical analysis of small signals. Phys. Rev. D 57, 3873–3889 (1998).

[b50] Clayton D. D. The role of radioactive isotopes in astrophysics. Lect. Notes Phys. 812, 25–79 (2011).

[b51] Lodders K. Solar system abundances and condensation temperatures of the elements. Astrophys. J. 591, 1220–1247 (2003).

[b52] Altobelli N. . Cassini between Venus and Earth: detection of interstellar dust. J. Geophys. Res. 108, 8032 (2003).

[b53] Westphal A. J. . Evidence for interstellar origin of seven dust particles collected by the Stardust spacecraft. Science 345, 786–791 (2014). 25124433 10.1126/science.1252496

[b54] Donelly J. . Actinide and ultra-heavy abundances in the local galactic cosmic rays: an analysis of the results from the *LDEF* ultra-heavy cosmic ray experiment. Astrophys. J. 747, 40 (2012).

[b55] Wallner C. . Development of a very sensitive AMS method for the detection of supernova-produced long-lived actinide nuclei in terrestrial archives. Nucl. Instr. Meth. B 172, 333–337 (2000).

[b56] Paul M. . An upper limit to interstellar ^244^Pu abundance as deduced from radiochemical search in deep-sea sediment: an account. J. Radioanal. Nucl. Chem. 272, 243–245 (2007).

[b57] Ferrier K. The interstellar environment of our galaxy. Rev. Mod. Phys. 73, 1031 (2001).

[b58] Breitschwerdt D., de Avillez M. A., Feige J. & Dettbarn C. Interstellar medium simulations. Astron. Nachr. 333, 486–496 (2012).

[b59] Cox D. P. & Anderson P. R. Extended adiabatic blast waves and a model of the soft x-ray background. Astrophys. J. 253, 268–289 (1982).

[b60] Fry B. J., Fields B. D. & Ellis J. R. Astrophysical shrapnel: discriminating among extra-solar sources of live radioactive isotopes. Preprint at http://arxiv.org/abs/arXiv:1405.4310 (2014).

[b61] Stuart F. M. & Lee M. R. Micrometeorites and extraterrestrial He in a ferromanganese crust from the Pacific Ocean. Chem. Geol. 322–323, 209–214 (2012).

[b62] Basu S., Stuart F. M., Schnabel C. & Klemm V. Galactic-cosmic-ray-produced ^3^He in a ferromanganese crust: any supernova ^60^Fe excess on Earth? Phys. Rev. Lett. 98, 141103 (2007). 17501264 10.1103/PhysRevLett.98.141103

[b63] Wallner A. .^60^Fe at the ANU – —search for a live supernova signature and a new half-life measurement. In: *Presentation at the 13th Intern. Conf. on Accelerator Mass Spectrometry*, 24−29 August 2014, 36 (Aix en Provence, France) (2014).

[b64] Ludwig P. . Search for supernova produced ^60^Fe in Earth's microfossil record. In: *Presentation at the 13th International Conference on Accelerator Mass Spectrometry*, 24−29 August 2014, 37 (Aix en Provence, France) (2014).

[b65] Fimiani L. . In Lunar and Planetary Science Conference, 18-22 March 2013, Vol. **45**, 1778 (Woodlands, TX, USA, (2014).

[b66] Feige J. . The search for supernova-produced radionuclides in terrestrial deep-sea archives. Publ. Astron. Soc. Aust. 29, 109–111 (2012).

[b67] Wallner A. Nuclear astrophysics and AMS—probing nucleosynthesis in the lab. Nucl. Instr. Meth. B 268, 1277–1282 (2010).

[b68] Wallner A. . A novel method to study neutron capture of ^235^U and ^238^U simultaneously at keV energies. Phys. Rev. Lett. 112, 192501 (2014). 24877933 10.1103/PhysRevLett.112.192501

[b69] Steier P. . Analysis and application of heavy isotopes in the environment’. Nucl. Instr. Meth. B 268, 1045 (2010).

[b70] Dauphas N. Multiple sources or late injection of short-lived *r*-nuclides in the early solar system? Nucl. Phys. A. 758, 757c–760c (2005).

