# Supplementary material for: Abundance of live 244Pu in deep-sea reservoirs on Earth points to rarity of actinide nucleosynthesis
Source: Nat Commun. 2015 Jan 20;6:5956. doi: 10.1038/ncomms6956 (PMC4309418; doi:10.1038/ncomms6956)
Supplement: Supplementary Information — Supplementary Figure 1, Supplementary Tables 1-4 and Supplementary References. [file ncomms6956-s1.pdf]

Supplementary Figure 1: Individual data for the ^{244}Pu flux into the crust as measured with AMS for the various subsamples. For the calculation of the ISM flux at Earth orbit these data were adjusted to account for the incorporation efficiency (21±5%) and also multiplied by a factor of 4 to account for the four times larger surface of the Earth compared to its cross section. This plot demonstrates that section D (in particular samples D3 and D4) dominate the final result due to their highest measurement efficiency.

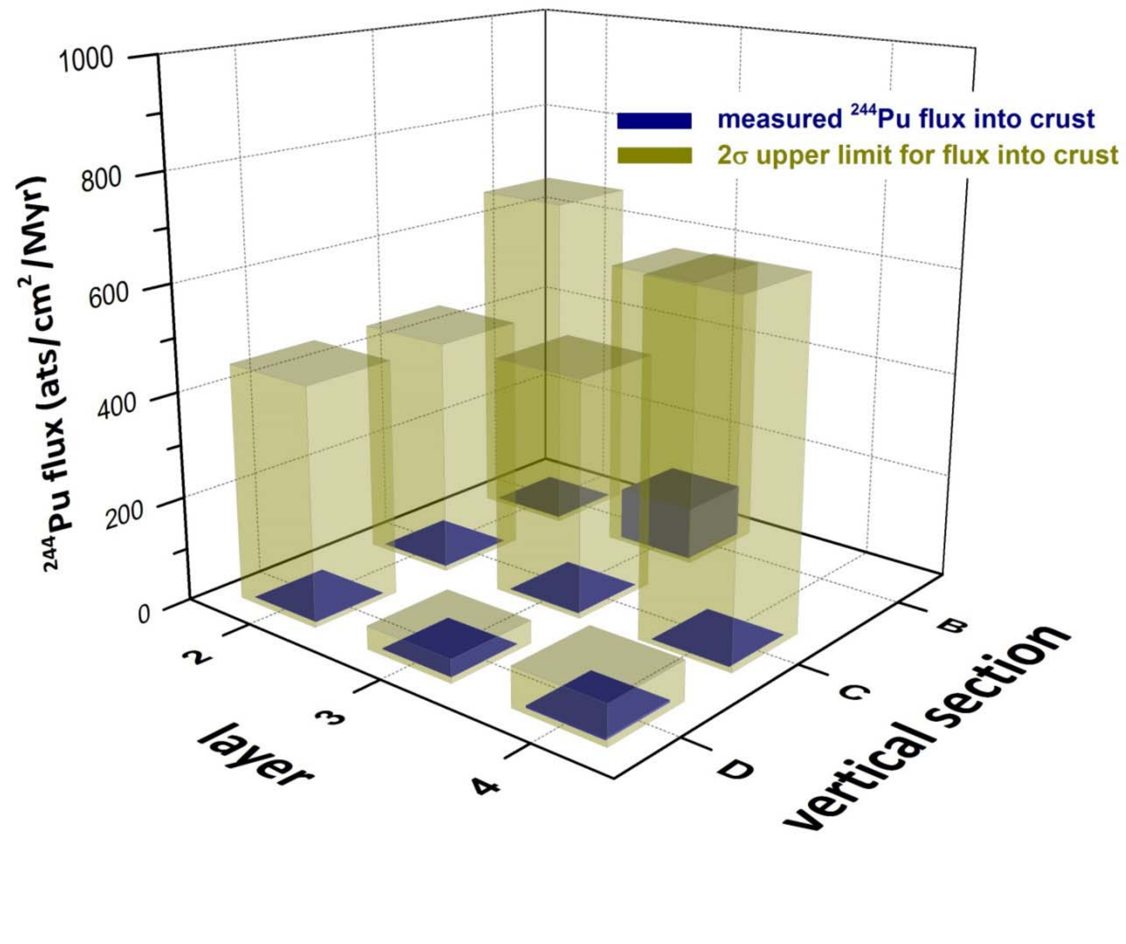

Supplementary Table 1: 237KD (VA13/2) deep-sea crust measurement: detailed data for the surface layer 1 (anthropogenic Pu) and the hydrothermal blank sample

		**Surface Layer 1**				**blank**
	time period	0 – 0.5 My				**--**
		contains anthropogenic Pu (top 1 mm)				**hydrothermal**
						**~ 100 cm^2**
	subsample	B1	C1	D1	**Total**	**X**
	mass (g)	32	20	28	**80**	**364**
	time period (My)	0 – 0.5				--
Particle detector events -RUN-1	*^{236}Pu events (11.1%)*		1511	608	**2119**	**3094**
	^{244}Pu events (83.3%)		2	2	**4**	**0**
	^{239}Pu events (5.6%)		3940	999	**4939**	**154**
Particle detector events -RUN-2	*^{236}Pu events (10.5%)*	2602			**2602**	
	^{244}Pu events (79.0%)	12			**12**	
	^{239}Pu events (10.5%)	11978			**11978**	
	total meas. eff. (10^{-4})	0.82	0.45	0.18	**<0.51>**	**0.93**
	measuring time	3.8 h	3.8 h	2.6 h		**3.4 h**
	^{236}Pu atoms spike	3×10^8	3×10^8	3×10^8		**3×10^8**
	^{244}Pu atoms (10^4)	18.5	5.3	13.3	**37.1**	**< 1.7**
	^{239}Pu atoms (10^8)	13.9	15.6	9.9	**39.4**	**0.3**
	^{244}Pu/^{239}Pu (10^{-4})	1.3	0.3	1.3	**1.0±0.3**	**--**

The total measurement efficiency (eff.) is calculated from the total number of ^{236}Pu registered, normalized by the time fraction of ^{236}Pu AMS counting (RUN-1 11.1% and RUN-2 10.5%, respectively) and divided by the number of ^{236}Pu atoms added as spike to the sample (3×10^8 atoms each).

The ^{244}Pu atoms per sample are calculated from the number of ^{244}Pu events registered with the particle detector, scaled by the time fraction of AMS ^{244}Pu counting (83.3% RUN-1 and 79.0% RUN-2) and normalized with the measurement efficiency; ditto for ^{239}Pu atoms per sample. The ^{239}Pu detector events were corrected for a well-known contribution when adding the ^{236}Pu spike which contains also ^{239}Pu (see Supplementary Table 4: Reference samples).

Supplementary Table 2: detailed data for 237KD (VA13/2) deep-sea crust measurement – samples used for the search of extraterrestrial ^{244}Pu

		Section B				Section C			Section D				total - with anthropogenic ^{244}Pu correction
	time period	0.5 – 12 My				0.5 – 25 My			0.5 – 25 My				0.5 – 25 My
	sample area	85.3 cm^2				70 cm^2			72.2 cm^2				<182> cm^2
	time×area (cm^2×My)	981				1715			1769				4465
	Subsample	B2	B3	B4	**total-B**	C2&C3	C4	**total-C**	D2	D3	D4	**total-D**	**B + C + D**
	mass (g)	133	312	-	**445**	447	250	**697**	116	286	364	**766**	**1908**
	time period (My)	0.5-5	5 - 12		**11.5 My**	0.5-12	12 - 25	**24.5 My**	0.5-5	5 – 12	12 – 25	**24.5 My**	**24.5 My**
RUN-1	*^{236}Pu det. events (t_{236}=11.1%)*		586			348	184						
	^{244}Pu det. events (t_{244}=83.3%)		1		**1**	0	0	**0**					**1^{+3}_{-1}**
	^{239}Pu det. events (t_{239}=5.6%)		63			31	49						
RUN-2	*^{236}Pu det. events (t_{236}=10.5%)*	464							829	4506	2435	**7770**	
	^{244}Pu det. events (t_{244}=79.0%)	0							0	0	1	**1**	**1^{+3}_{-1}**
	^{239}Pu det. events (t_{239}=10.5%)	95							99	1010	1162	**2271**	
sample data	*total eff. ε (10^{-4})*	0.15	0.18		**<0.17>**	0.10	0.06	**<0.08>**	0.26	1.43	0.77	**<0.868>**	**<0.42>**
	^{244}Pu det. Events	0	1		**1**	0	0	**0**	0	0	1	**1**	**$2^{+4.7}_{-1.6}$**
	time×area×eff. ×t_{244} (cm^{2}My)				**0.014**			**0.012**				**0.124**	**0.151**
	AMS counting time	16.5h	2.3h	-	**18.8 h**	2.4h	2.1 h	**4.5 h**	14.6h	18.8h	15 h	**48.4 h**	**71.7 h**
^{244}Pu fluence into crust (*at/cm^2*)		<3,000	780	-	**850**	< 5,100	< 8,600	**< 6,400**	< 2,000	< 290	230	**200**	**320^{+760}_{-260}**
^{244}Pu flux into crust (*at/cm^2/My*)		< 660	110		**74**	< 450	< 660	**< 260**	< 450	< 42	18	**8**	**13^{+31}_{-11}**
**^{244}Pu ISM flux *(at/cm^2/My) at 1 AU***					**1,400**			**< 4,900**				**155**	**250^{+590}_{-205}**

Uncertainties given are based on statistical analysis of low signals[1]. The time fraction of AMS counting of the various isotopes (detector events) is given in parenthesis in column two. Numbers given in angle brackets (e.g. <182> cm^2) denote averaged values. The total efficiency was calculated as described in Table 3 (with a ^{236}Pu spike per sample of 3×10^8 atoms). The ^{239}Pu concentration (^{239}Pu at per gram) in these older crust samples was found typically a factor of ~100 lower ($(2–4)\times10^5$ ^{239}Pu at/g crust) than measured for the surface samples 5×10^7 ^{239}Pu at/g crust). We attribute this measured ^{239}Pu concentration rather to leaking ^{238}U atoms mimicking ^{239}Pu because presence of natural ^{239}Pu is negligible in these older layers. The ^{244}Pu atoms per sample were calculated from the (upper limits of the) particle detector events, the total efficiency and by taking into account the time fraction of ^{244}Pu counting (83.3% and 79% for RUN-1 and RUN-2, respectively). Anthropogenic ^{244}Pu background is <<1 in all cases.

The ^{244}Pu ISM flux was calculated from the flux into the crust, the incorporation efficiency of 21% (see Table 3) and multiplied by a factor of 4 to account for the 4 times larger Earth surface compared to its cross section (i.e. assuming a unidirectional and homogeneous ISM flux relative to the solar system).

The measured data for the ^{244}Pu flux for the individual subsamples are plotted in Supplementary Figure 1.

Supplementary Table 3: Detailed data for the deep-sea sediment samples and two ^{242}Pu reference samples

	ref-1	ref-2	TR_53-138	TR_149-217	**total sediment**
	^{242}Pu – reference		**deep-sea sediment**		
mass (g)	--	--	57.62 g	43.39 g	**101.0**
AMS counting time (h)	9.6	9.6	11.2	15.7	**26.9 h**
spike (^{242}Pu at):	2.47×10^{8}	2.47×10^{8}	2.47×10^{8}	2.47×10^{8}	**4.94×10^{8}**
measurement efficiency (10^{-4})	0.58	0.84	0.94	2.53	**<1.73>**
^{242}Pu detector events (7.1%)	1,020	1,481	1,640	4,437	**6,077**
^{244}Pu detector events (89.3%)	0	0	0	1	**1**
^{239}Pu detector events (3.6%)	1	1	29	24	**53**
^{244}Pu per sample (10^{4})			< 4	0.6	**$0.6^{+2.4}_{-0.6}$**
^{244}Pu at / g sediment	--	--	< 730	134	**59^{+236}_{-56}**
^{244}Pu / cm^2 (4.9 cm^2)	--	--	< 8,300	1,200	**$1{,}300^{+5{,}200}_{-1{,}235}$**
^{244}Pu /cm^2 / My flux into sediment	--	--			**$750^{+3{,}000}_{-710}$**
**^{244}Pu ISM flux *(at/cm^2/My)***					**$3{,}000^{+12{,}000}_{-2{,}850}$**

The time fraction of AMS counting of the various isotopes is given in parenthesis in the first column next to the label for the '^{242,244,239}Pu detector events' (7.1, 89.3 and 3.6%, respectively). The upper limits given are purely statistical[1] and represent 95% confidence levels. The total efficiency was calculated with a ^{242}Pu spike per sample of 2.47×10^{8} atoms; the anthropogenic ^{244}Pu detector event rate was calculated from the ^{239}Pu count rate and the measured ^{244}Pu/^{239}Pu isotope ratio of the surface sample (1.0×10^{-4}).

Note: Raisbeck et al.[2] quote a limit of $<10^{4}$ ^{244}Pu at/g of sediment and Paul et al.[3] quote a limit of $<10^{3}$ at/g sediment (90% confidence level).

The ^{244}Pu ISM flux is calculated from the flux into the sediment, the incorporation efficiency of 100% and multiplied by a factor of 4 to account for the 4 times larger Earth surface compared to its cross section (i.e. assuming a unidirectional and homogeneous ISM flux relative to the solar system).

Supplementary Table 4: Reference samples (^{236}Pu and ^{244}Pu/^{242}Pu) for the crust samples; and a surface sample from a Mediterranean sediment

	Spike-1	Spike-2	ST7			Pu-100_1	Pu-30_1	Pu-30_2
	^{236}Pu – reference		Mediterranean sediment - surface			^{244}Pu – reference 1	^{244}Pu – reference 2	^{244}Pu – reference 2
Mass (g)			5g					
T_M (h)	9.7	17.3	24.7					
^{236}Pu detector events (time fraction)	47,754 (18.6%)	30,395 (9.8%)	447 (17.4%)		^{236}Pu evts (18.2%)	18	6	6
^{244}Pu detector events (time fraction)	0 (69.8%)	0 (73.6%)	12 (65.2%)		^{244}Pu evts (36.4%)	3,529	2,112	2,225
^{239}Pu detector events (time fraction)	6,866 (7.0%)	11,693 (9.8%)	14,644 (6.5%)		^{239}Pu evts (9.1%)	0	1	3
^{244}Pu/^{239}Pu	$<1.9\times10^{-5}$	$<1.5\times10^{-5}$	$(8\pm2)\times10^{-5}$		^{244}Pu/^{239}Pu	--	--	--
^{242}Pu detector events (time fraction)	29 (4.7%)	20 (6.7%)	2,424 (10.9%)		^{242}Pu evts (36.4%)	28,133	54,009	57,938
^{239}Pu/^{236}Pu[*]	0.382	0.385	--		^{244}Pu/^{242}Pu	0.125	0.039	0.038
total efficiency (10^{-4})	> 0.[‡]	> 1.0[‡]	0.89			--	--	--
spike (ats):	3×10^9	3×10^9	2.5×10^8					

Spike test samples for ^{236}Pu detection, sediment surface sample from the Mediterranean sea-floor (ST7) for ^{242}Pu detection, and reference materials with known ^{244}Pu/^{242}Pu isotope ratios (the nominal value of Pu-30 is one third of Pu-100 which was reproduced in the AMS measurement).

[*] the ^{236}Pu spike was found to contain ^{239}Pu with a ratio of ^{236}Pu/^{239}Pu = (0.384±0.003); this ^{239}Pu contribution from the spike was corrected for in the crust samples prior to estimating anthropogenic ^{244}Pu on the basis of ^{239}Pu presence in deep crust layers.

[‡] the sample material in the AMS measurement was not fully consumed for the two ^{236}Pu reference samples therefore only lower limits are given.

Supplementary References:

1. Feldman G.J. & Cousins, R.D., Unified approach to the classical statistical analysis of small signals. *Phys. Rev. D* **57**, 3873-3889 (1998).

2. Raisbeck, G. *et al.*, A search for supernova produced ^{244}Pu in a marine sediment, *Nucl. Instr. and Meth. B* **259**, 673-676 (2007).

3. Paul, M. et al. An upper limit to interstellar ^{244}Pu abundance as deduced from radiochemical search in deep-sea sediment: An account. *J. of Radioanal. and Nucl. Chem.* **272**, 243–245 (2007).
